# Global Dual Incidence‐Mortality Patterns of Prostate Cancer Across SDI Regions, 1990–2021: A Cross‐Sectional Study Based on Global Burden of Disease 2021

**DOI:** 10.1002/hsr2.72663

**Published:** 2026-07-28

**Authors:** Zhiping Ma, Cuicui Wang, Qin Zhang, Zhongyu Lu, Dongsheng Chen, Xing Zhang, Yan Wang

**Affiliations:** ^1^ Department of Pathology, Xinjiang Uygur Autonomous Region The First Affiliated Hospital of Xinjiang Medical University Urumqi China; ^2^ Department of Pathology, Xinjiang Uygur Autonomous Region Xinjiang Medical University Affiliated Tumor Hospital Urumqi China; ^3^ State Key Laboratory of Neurology and Oncology Drug Development, Jiangsu Simcere Diagnostics Co., Ltd. Nanjing Simcere Medical Laboratory Science Co., Ltd. Nanjing China; ^4^ Department of Oncology General Hospital of Ningxia Medical University Yinchuan China

## Abstract

**Background:**

To facilitate the development of precision prevention and control measures, this research offers a comprehensive evaluation of the global prostate cancer landscape, with a specific focus on its incidence, mortality rates, and underlying risk factors.

**Methods:**

We retrieved data on prostate cancer incidence, fatalities, and disability‐adjusted life‐years (DALYs) across 204 nations and territories from the Global Burden of Disease (GBD 2021) study. Trends were assessed via estimated annual percentage changes (EAPC), with further stratification by age and the socio‐demographic index (SDI).

**Results:**

Globally, prostate cancer cases and deaths reached 1.32 million and 432,000 in 2021, marking a substantial rise of 162% and 104% compared to 1990 levels. While the global age‐standardized incidence rate (ASIR) remained stable, it rose significantly in low‐SDI regions (EAPC = 0.69%) and declined in high‐SDI regions (EAPC = −0.36%). The global age‐standardized mortality rate (ASMR) decreased (EAPC = −1.05%), and the age‐standardized DALY rate showed a similar downward trend, with higher rates in low‐SDI regions. Mortality in low‐SDI regions (16.8/100,000) surpassed that in high‐SDI regions (15.35/100,000). Incidence peaked at ages 70–74, whereas mortality peaked at ≥ 85 years. Tobacco control and dietary interventions are beneficial, but their population impact is modest in low‐SDI regions.

**Conclusion:**

Prostate cancer burden follows a dual pattern: ASIR increases with SDI, while ASMR and DALY rates decrease. High‐SDI regions have reduced mortality through screening/treatment advances, whereas low‐SDI regions face rising incidence and persistently high mortality. Although tobacco control and diet help, their attributable fractions are modest; health‑system strengthening for early detection and treatment access is the priority in low‑SDI settings.

## 1 Introduction

Globally, prostate cancer remains the most common cancer diagnosed in the male demographic, while also serving as the second most frequent source of mortality due to cancer in men [1]. Its insidious nature often leads to late‐stage diagnosis, reducing treatment efficacy and quality of life [2]. Clinical management faces challenges including treatment selection, drug resistance, and high costs [3]. Furthermore, unequal distribution of medical resources contributes to marked disparities in screening and treatment, exacerbating the disease burden [4].

Although a large number of studies have been conducted, there are still important areas that are not fully understood regarding the worldwide patterns of prostate cancer incidence and death [5, 6, 7]. To develop more precise screening and treatment strategies, further investigation is warranted into the geographical distribution, temporal trends, and the influence of age on the incidence and mortality rates of prostate cancer. It is therefore worthwhile to provide a comprehensive understanding of the global epidemiological burden of prostate cancer, as well as its variations across different regions and age groups, utilizing the Global Burden of Disease (GBD) database. The GBD database offers a thorough evaluation of diseases, injuries, and risk factors on a global scale, with its most recent data facilitating the quantification of health losses across diverse regions and timeframes while mitigating discrepancies [8].

The primary objective of this study is to analyze the epidemiological characteristics of prostate cancer through the GBD database, specifically to investigate trends in incidence and mortality across different socio‐demographic index (SDI) levels, and to explore risk factors of prostate cancer by SDI strata, elucidate regional and age‐related differences, and discuss the public health and clinical implications of these findings. To conclude, the current investigation aims to establish a robust framework of evidence for the development of improved measures for prevention to decrease the frequency and death rates of prostate cancer.

## 2 Methods

### 2.1 Population Data Source

For this analysis of the GBD 2021, we obtained de‐identified data from the Global Health Data Exchange platform, managed by the Institute for Health Metrics and Evaluation at the University of Washington. The data are publicly available at https://vizhub.healthdata.org/gbd-results [9, 10]. GBD 2021 findings equip policymakers, public‐health professionals, and researchers with critical evidence to identify health disparities within and across populations, monitor long‐term trends, measure health progress, and develop strategies to address post‐COVID‐19 health inequities [11]. Since this study utilized secondary data compiled by country and at the global scale, formal institutional ethics review was not required.

The study population comprised individuals diagnosed with prostate cancer. For GBD 2021, prostate cancer was identified using the International Classification of Diseases (ICD), Ninth Revision (ICD‐9) and Tenth Revision (ICD‐10) codes (ICD‐10: C61–C61.9; ICD‐9: 185–185.9, V10.46, V16.42, V76.44). Estimates of incidence, prevalence, mortality, and disability‐adjusted life‐years (DALYs) were derived from household surveys, censuses, vital registration, and other health‐related data sources. Cause‐specific mortality was calculated using the Cause of Death Ensemble model, with 95% uncertainty intervals (UIs) provided [6]. Disability weights (DWs) quantify the severity of health loss or non‐fatal disability; years lived with disability (YLDs) = number of prevalent cases × duration until remission or death × DW. Years of life lost (YLLs) = number of deaths × standard life expectancy at age, location, and year. DALYs = YLLs + YLDs [12]. We estimated age‐standardized incidence rates (ASIR), age‐standardized mortality rates (ASMR), and age‐standardized DALY rates (ASDR) for the 34 cancers classified as Level 3 causes in the GBD framework.

This study provides a comprehensive assessment spanning 1990–2021 across five SDI levels, 21 GBD regions, 204 countries and territories, 371 diseases and injuries, and 88 risk factors (Figure S1). The SDI is a composite indicator of national development that incorporates per‐capita income, mean years of schooling, and the fertility rate among women under 25; it ranges from 0 to 1. We classified locations as high (0.805129–1), high‐middle (0.689504–0.805129), middle (0.607679–0.689504), low‐middle (0.454743–0.607679), or low (0–0.454743) SDI [13]. Using the GBD comparative risk assessment framework, burden attributable to risk factors was estimated and organized into four hierarchical levels; this study focused on Level 4 risks [14]. All analyses were stratified across nine age groups: 45–49, 50–54, 55–59, 60–64, 65–69, 70–74, 75–79, 80–84, and ≥ 85 years.

### 2.2 Statistical Analysis

Descriptive analyses were used to present the global burden of prostate cancer from 1990 to 2021, comparing age‐standardized prevalence (per 100,000), age‐standardized mortality (per 100,000), and their estimated annual percentage changes (EAPC) across SDI levels. Temporal trends were evaluated with EAPC and 95% confidence intervals (CIs) using the linear regression model as follows: *Y* = *α* + *βX* + *ε*, where *Y* is the natural logarithm of the age‐standardized rate (ASR), *X* is calendar year, and ε is the error term; EAPC = 100 × [exp(β) − 1] [15]. Within the GBD framework, burden estimates for prostate cancer are reported as means with 95% UIs; the burden attributable to each risk factor is expressed as a percentage of total deaths (%) and DALYs (%) with 95% UIs. The 95% UIs were derived from 1000 draws, with lower and upper bounds taken as the 25th and 975th ordered values. We examined correlations between prostate cancer risk factors and SDI, incidence and mortality rates as well as EAPC by age group and SDI level, and performed a period analysis of incidence. Joinpoint regression [16] was performed to characterize temporal trends in age‑standardized rates of prostate cancer incidence, mortality, and disability‑adjusted life years (DALYs) from 1990 to 2021. A log‑linear model was employed, and heteroscedasticity was addressed by incorporating the standard errors of each rate as weights. The minimum number of joinpoints was set to 0 and the maximum to 6. The optimal number of joinpoints was selected via permutation tests (4500 permutations). The annual percent change (APC) for each segment was calculated along with their 95% CIs. All statistical tests were two‐sided, with a priori significance level set at *α* = 0.05. Analyses were performed using R v4.5.0 and Joinpoint Regression Program version 5.2.0.

## 3 Results

### 3.1 Cancer Burden and SDI

An estimated 13.24 × 10^5^ (95% UI: 12.17–14.00) new prostate cancer cases occurred worldwide in 2021, representing a 162% increase from 5.06 × 10^5^ cases (95% UI: 4.81–5.25) in 1990 (Table 1). Over the same period, the ASIR declined slightly, from 32.64 per 100,000 (95% UI: 30.86–33.86) to 34.05 per 100,000 (95% UI: 31.27–36.00), yielding an EAPC of −0.06% (95% CI: 0.20%–0.08%). After adjusting for population growth and aging, the underlying risk has remained largely unchanged globally. However, this overall stability masks substantial regional heterogeneity. From 1990 to 2021, ASIR in high‐SDI regions initially rose and then stabilized; high‐middle‐SDI regions remained relatively stable, whereas middle‐, low‐middle‐, and low‐SDI regions exhibited upward trends (Figure S2A). In 2021, ASIR in high‐SDI regions (70.92 [95% UI: 66.29–74.22] per 100,000) and high‐middle‐SDI regions (30.36 [95% UI: 26.96–33.04] per 100,000) far exceeded the global average (34.05 [95% UI: 31.27–36] per 100,000) and the rate in low‐SDI regions (18.14 [95% UI: 11.84–22.34] per 100,000). Low‐SDI had the lowest point estimate but the widest UI, reflecting sparse data; in contrast, the UI for high‐SDI was narrow, indicating high precision. Between 1990 and 2021, significant ASIR increases were observed in middle‐SDI (EAPC 1.23%), low‐middle‐SDI (EAPC 1.61%), and low‐SDI (EAPC 0.69%) regions. ASIR also rose significantly in high‐middle‐SDI regions (EAPC 0.83%), whereas high‐SDI regions experienced a significant decline (EAPC –0.36%).

**Table 1:** Incidence, mortality, and EAPC of prostate cancer from 1990 to 2021 in global and 5 SDI levels.

Location	Incidence					Mortality				
	1990		2021		EAPC	1990		2021		EAPC
	Incidence (×105,95% UI)	ASIR (1/100,000, 95% UI)	Incidence (×105,95% UI)	ASIR (1/100,000, 95% UI)		Mortality (×105,95% UI)	ASMR (1/100,000, 95% UI)	Mortality (×105,95% UI)	ASMR (1/100,000, 95% UI)	
Global	5.06 (4.81–5.25)	32.64 (30.86–33.86)	13.24 (12.17–14)	34.05 (31.27–36)	−0.06 (−0.2 to 0.08)	2.12 (1.94–2.24)	16.35 (15.02–17.28)	4.32 (3.82–4.64)	12.63 (11.16–13.55)	−1.05 (−1.14 to −0.95)
High SDI	3.39 (3.25–3.49)	73.74 (70.21–75.85)	6.95 (6.47–7.28)	70.92 (66.29–74.22)	−0.36 (−0.52 to −0.21)	1.05 (0.99–1.08)	25.71 (24.06–26.61)	1.54 (1.39–1.64)	15.35 (13.8–16.27)	−2.02 (−2.14 to −1.91)
High‐middle SDI	0.84 (0.79–0.88)	22.76 (21.31–23.86)	2.68 (2.38–2.92)	30.36 (26.96–33.04)	0.83 (0.63–1.03)	0.43 (0.41–0.46)	14.25 (13.2–15.09)	0.9 (0.8–0.99)	11.73 (10.33–12.86)	−0.79 (−0.97 to −0.62)
Middle SDI	0.47 (0.4–0.52)	12.49 (10.8–13.93)	2.28 (1.95–2.59)	19.43 (16.51–22.07)	1.23 (1.05–1.41)	0.31 (0.26–0.36)	10.14 (8.57–11.64)	1 (0.83–1.16)	10.03 (8.3–11.6)	−0.17 (−0.35 to 0.01)
Low‐middle SDI	0.23 (0.18–0.27)	9.46 (7.46–11.07)	0.96 (0.8–1.11)	15.9 (13.29–18.51)	1.61 (1.48–1.74)	0.19 (0.15–0.23)	8.77 (6.78–10.39)	0.58 (0.47–0.68)	10.92 (9.02–12.93)	0.68 (0.55–0.81)
Low SDI	0.13 (0.08‐0.16)	14.5 (9.59–18.27)	0.36 (0.23–0.44)	18.14 (11.84–22.34)	0.69 (0.62–0.75)	0.12 (0.08–0.15)	15.02 (9.92–19)	0.29 (0.19–0.36)	16.8 (10.86–20.59)	0.36 (0.29–0.42)

Abbreviations: ASIR, age‐standardized incidence rate; ASMR, age‐standardized mortality rate; EAPC, estimated annual percentage change; CI, confidence interval; UI, uncertainty intervals.

In 2021, global prostate cancer deaths reached 4.32 × 10^5^ (95% UI: 3.82–4.64), a 104% increase from 2.12 × 10^5^ deaths (95% UI: 1.94–2.24) in 1990. The ASMR declined markedly, from 16.35 per 100,000 (95% UI: 15.02–17.28) to 12.63 per 100,000 (95% UI: 11.16–13.55), corresponding to an EAPC of −1.05% (95% CI: −1.14% to −0.95%), indicating steady global improvement in prostate cancer‐related mortality risk. Nevertheless, trends differed across SDI levels: ASMR declined in high‐, high‐middle‐, and middle‐SDI levels, whereas low‐middle‐ and low‐SDI regions experienced slight increases (Figure S2B). In 2021, ASMR in high‐SDI regions (15.35 per 100,000) remained above the global average (12.63 per 100,000), whereas low‐SDI regions exhibited the highest ASMR at 16.80 per 100,000. The magnitude of ASMR decline varied substantially: high‐SDI regions showed the sharpest reduction (EAPC −2.02%), followed by high‐middle‐SDI (EAPC −0.79%) and middle‐SDI (EAPC −0.17%) levels. ASMR in low‐middle‐SDI levels remained almost stable (EAPC 0.68%), while low‐SDI regions showed minimal change (EAPC 0.36%). These divergent trajectories resulted in low‐SDI level surpassing high‐SDI in 2021 (16.80 vs. 15.35 per 100,000), despite having the fewest absolute cases. Thus, incidence and mortality move in opposite directions across SDI strata, a dual pattern that reflects differences in detection and treatment capacity rather than a uniform burden gradient. Moreover, both ASIR (*r* = −0.86, *p* = 0.03) and the ASDR (*r* = −0.87, *p* = 0.03) were significantly negatively correlated with EAPC (Figure S3). These negative correlations suggest that regions with higher baseline rates (primarily high‐SDI) have transitioned toward burden stabilization or reduction, while regions with lower baseline rates (primarily low‐SDI) face accelerating rates, consistent with the diffusion of screening and diagnostic capacity over time.

### 3.2 Period Analysis of Global Prostate Cancer Incidence

**Figure 1:**
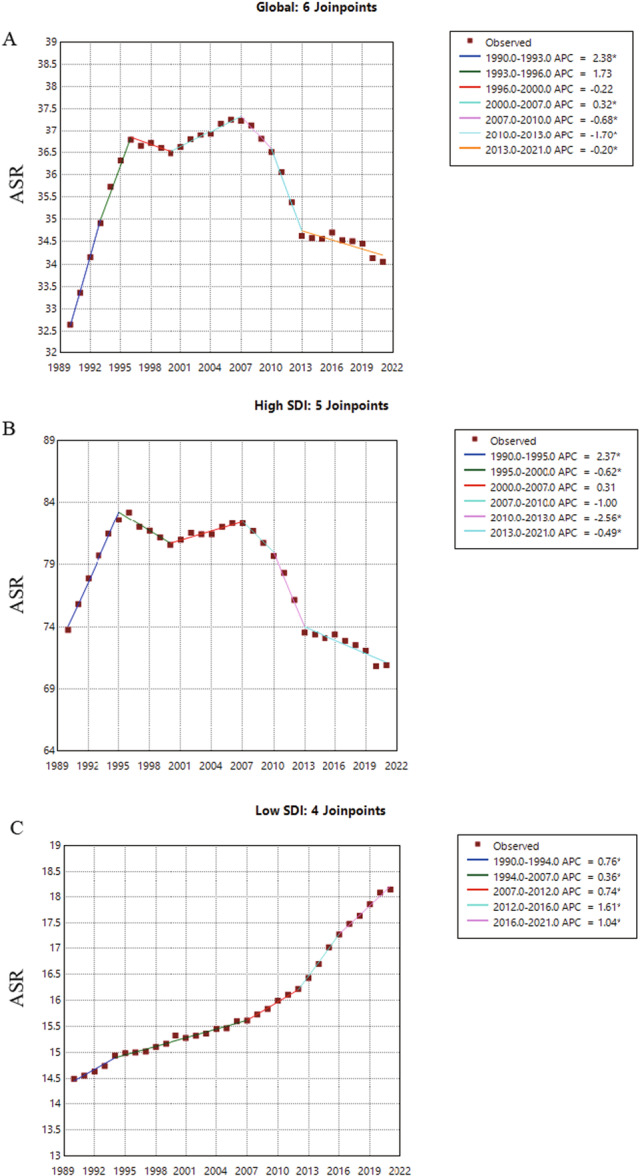
Joinpoint regression analysis of ASR in prostate cancer worldwide from 1990 to 2021. (A) Global trends in ASIR, showing six inflection points with APC annotated for each segment. (B) High‐SDI regions exhibited a rapid rise during 1990–1995. (C) Low‐SDI regions showed a slow, steady increase throughout the period, with wider uncertainty bands reflecting sparser data and higher estimation uncertainty compared to high‐SDI regions.

Joinpoint regression of ASRs from 1990 to 2021 identified 6 inflection points worldwide (Figure 1A). ASRs rose between 1990 and 1996 (APC 1990–1993 = 2.38 [95% CI: 2.11–2.71]; APC 1993–1996 = 1.73 [95% CI: −0.17–1.92]), fluctuated slightly from 1997 to 2001 (APC = −0.22 [95% CI: −0.45–0.13]), and peaked in 2005 at 37.2%. A sustained decline began in 2008; by 2021, the ASR had fallen below the 1995 level (34.1% vs. 36.3%). High‐SDI level mirrored the global pattern (Figure 1B). ASRs climbed rapidly during 1990–1995 (APC = 2.37 [95% CI: 2.17–2.59]), then declined after 1997 with minor fluctuations to 70.9% in 2021, below the 1990 value of 73.7%. By contrast, low‐SDI exhibited a slow, steady increase (Figure 1C) but carried the greatest data uncertainty (standard error [SE], consistently > 2.2), peaking at SE = 2.68 in 2021.

### 3.3 National Cancer Burden

**Figure 2:**
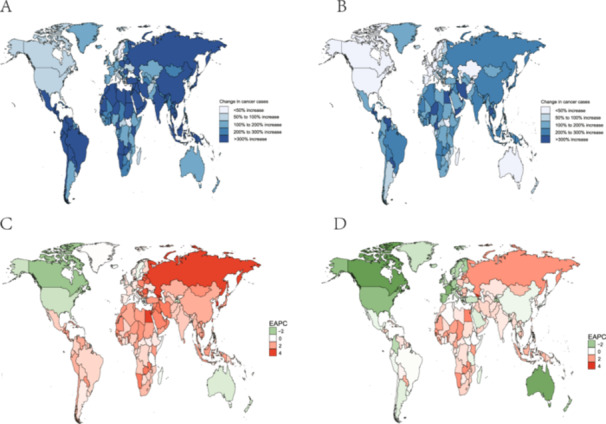
Global geographic patterns of prostate cancer burden change from 1990 to 2021. (A) Percent change in incident cases. (B) Percent change in death cases. Color intensity indicates magnitude of change in both A and B. (C) EAPC in ASIR. (D) EAPC in ASMR. Green indicates declining rates, yellow‐to‐red indicates increasing rates, with deepest red showing steepest rises in (C, D).

When evaluating data for each country between 1990 and 2021, the most substantial proportional surges in prostate cancer cases and fatalities were documented in East Asia as well as the Middle East and North Africa. In contrast, the increases observed throughout North America and Western Europe North America and Western Europe were relatively modest (Figure 2A,B). The top four countries were identical for both metrics: Qatar (incidence increase/mortality increase: 1901.49%/528.26%), South Korea (1620.90%/562.93%), United Arab Emirates (1555.31%/537.27%), and Kuwait (1534.82%/1001.50%).

Examining EAPC over the same interval, rapid incidence increases, besides those in the Middle East & North Africa and East Asia, were also observed in Eastern Europe and sub‐Saharan Africa (Figure 2C,D). Among all countries, South Korea led with an incidence EAPC of 4.35 (95% CI 3.76–4.94), followed by Georgia 4.30 (95% CI 3.58–5.03), Estonia 4.14 (95% CI 3.43–4.85), and Egypt 4.07 (95% CI 3.82–4.33). For mortality EAPC, East Asia showed notable declines, while Eastern Europe remained high. Georgia again ranked first at 4.07 (95% CI 3.21–4.93), ahead of Egypt 2.58 (95% CI 2.25–2.92), Latvia 2.36 (95% CI 2.07–2.64), and Zambia 2.33 (95% CI 2.03–2.63). In contrast, Canada (incidence/mortality EAPC: −2.22/−3.15), the United States (−1.16/−2.32), and Australia (−0.82/−2.9) exhibited substantial decreases in both incidence and mortality.

### 3.4 Risk Factors Across SDI

**Figure 3:**
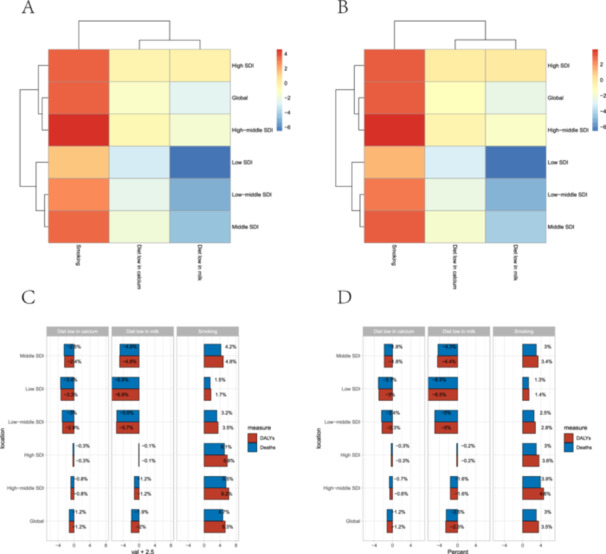
Attributable risk factors for prostate cancer DALYs and mortality across SDI levels in 1990 and 2021. (A) Heatmap of attributable DALYs (%) by risk factor and SDI level in 2021. (B) Heatmap of attributable deaths (%) by risk factor and SDI level in 2021. In both panels, smoking shows positive attribution (red), while low calcium and low dairy intake show negative attribution (blue). (C) Bar chart comparing 1990 attributable fractions for DALYs (red) and deaths (blue) across SDI levels. (D) Corresponding 2021 attributable fractions.

Next, we examined the attributable risk factors for prostate cancer and their variation across SDI levels. Smoking was the largest attributable risk factor among those examined for both DALYs and deaths, albeit with modest absolute fractions (Figure 3A,B). Low calcium and low dairy intake were associated with lower burdens. The pattern varied by SDI: smoking dominated in high‐ and high‐middle‐SDI regions, whereas the inverse associations with low calcium and low dairy intake were relatively more pronounced in low‐middle‐ and low‐SDI settings, despite their small absolute magnitudes.

Comparing risk‐factor contributions between 1990 (Figure 3C) and 2021 (Figure 3D), the burden attributable to smoking declined across all SDI levels. The reductions were most pronounced in high‐SDI (DALYs: 5.8%–3.6%; deaths: 5.1%–3%), high‐middle SDI (DALYs: 6.2%–4.6%; deaths: 5.5%–3.9%), and middle‐SDI (DALYs: 4.8%–3.4%; deaths: 4.2%–3%). These patterns indicate that attributable risk profiles differ substantially across development levels; the small magnitude of modifiable risk factors relative to total burden underscores the importance of healthcare access in explaining mortality disparities.

For dietary factors, the inverse associations of low calcium and low dairy intake remained modest in high‐ and high‐middle‐SDI regions in both 1990 and 2021 (high‐SDI 2021: low‐calcium DALYs −0.3%, deaths −0.3%; low‐dairy DALYs −0.2%, deaths −0.2%). In contrast, these dietary factors showed consistently stronger inverse associations in the remaining SDI levels, without appreciable attenuation over time. In 2021, low dairy intake remained particularly influential: middle‐SDI (DALYs −4.3%, deaths −4.4%), low‐middle SDI (DALYs −5%, deaths −5%), and low‐SDI (DALYs −6.5%, deaths −6.5%).

### 3.5 Incidence, Deaths and EAPC by Age Group and SDI Level

**Figure 4:**
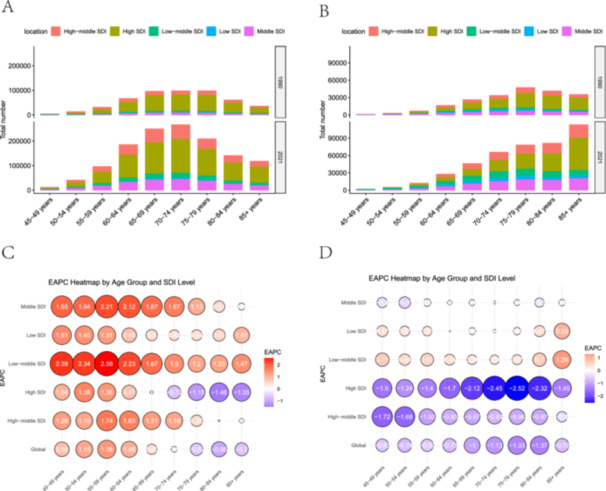
Age distribution and temporal trends in prostate cancer by SDI level, 1990–2021. (A) Stacked bar charts of incident cases by age group and SDI level, comparing 1990 (upper) and 2021 (lower). The incidence peak occurred at 70–74 years in both years, with high‐SDI regions (green) contributing the largest share across all ages in 2021. (B) Corresponding mortality distributions. Unlike 1990, 2021 mortality increased steadily with age, peaking at ≥ 85 years. (C) Bubble heatmap of incidence EAPC by age group and SDI level. EAPC increased in all age groups < 70 years across all SDI levels. (D) Mortality EAPC heatmap.

The age‑specific incidence patterns in 1990 and 2021 were almost identical, peaking at 70–74 years (Figure 4A). Unlike 1990, when the number of deaths began to decline after age 80, 2021 mortality increased steadily with age, reaching its maximum among those ≥ 85 years (Figure 4B). Compared with 2019, both incident cases and deaths increased in every age group in 2021. Although high‐SDI regions had the highest incidence proportion across all ages in 2021, their proportion of deaths relative to incidence was markedly lower.

Between 1990 and 2021, the overall incidence EAPC increased in all age groups below 70 years (45–49: 0.86%; 50–54: 1.18%; 55–59: 1.38%; 60–64: 1.06%; 65–69: 0.41%), and this rise was observed at every SDI level (Figure 4C). Among these younger age groups (45–49 years), low‐middle‐SDI recorded the highest EAPC. Conversely, high‐SDI experienced negative incidence EAPC in all four age groups ≥ 70 years: 70–74 (−0.72%), 75–79 (−1.15%), 80–84 (−1.48%), and ≥ 85 (−1.35%).

From 1990 to 2021, mortality EAPC declined across all age groups globally, a pattern mirrored by high‐, high‐middle‐, and middle‐SDI levels (Figure 4D). In contrast, low‐middle SDI showed rising mortality EAPC in every age group. Notably, low‐SDI regions had small declines at 65–69 years (−0.17%) and 70–74 years (−0.04%). The highest mortality EAPC were observed in the ≥ 85 group for both low‐middle‐SDI (0.99%) and low‐SDI (1.29%) regions.

## 4 Discussion

Leveraging GBD data, this study offers a comprehensive epidemiological appraisal of prostate cancer from 1990 to 2021. Despite global gains in some metrics, both incident cases and deaths continue to rise. The patterns of incidence, mortality, attributable risks, and age distribution differ markedly across SDI strata, showing a dual and opposing relationship: incidence rises with SDI while mortality falls with SDI. These findings provide a new evidence base for prostate cancer control strategies aimed at comprehensively reducing incidence and mortality.

This global trend masks a well‐described paradox in prostate cancer epidemiology: high‐SDI regions show high incidence but rapidly falling mortality, whereas low‐SDI regions show lower incidence but persistently high mortality. In high‐SDI settings, the widespread uptake of prostate‐specific antigen (PSA) screening has markedly increased early detection and lowered mortality [17], but it has also led to substantial overdiagnosis and overtreatment, with heightened economic costs. The 23‐year follow‐up of the European Randomized Study of Screening for Prostate Cancer (ERSPC) demonstrated that PSA‐based screening reduced prostate cancer mortality by 13% yet increased cumulative incidence by 30%, reflecting the extent of overdiagnosis [18]. Consequently, a high incidence profile is observed in high SDI regions, with an ASIR of 70.9 per 100,000 recorded in 2021. Furthermore, these regions have experienced a steep decrease in mortality (EAPC −2.02%), paralleling the successful outcomes of programs for cancer control implemented across Oceania, North America, and Northern and Western Europe [19, 20, 21]. Conversely, in low‐SDI settings, the absence of routine screening leads to underdiagnosis, and patients often present at advanced stages with limited access to curative therapies. A survey of Nigerian oncologists found that 36% of clinicians reported 61%‐80% of their patients presenting with advanced disease, and 87.5% indicated that few could afford enzalutamide, a second‐generation antiandrogen [22]. As a result, low‐SDI regions experience rising incidence (EAPC + 0.69%) with minimal mortality improvement (EAPC + 0.36%), culminating in the highest age‐standardized mortality rate (16.8 per 100,000 in 2021) despite the lowest absolute case counts [7]. These disparities highlight that the global prostate cancer burden is shaped not by a uniform risk gradient, but by profound differences in healthcare infrastructure, screening policies, and treatment accessibility across socioeconomic strata.

Global Joinpoint regression of ASR revealed a peak at 2005 followed by a sustained decline, with 2021 rates falling below those of 1995—a trend potentially related to changes in screening guidelines and practices, though ecological data preclude causal attribution [23]. In low‐SDI settings, ASR has risen slowly but with high uncertainty, underscoring the need to strengthen cancer‐registration systems. The fastest growth in incidence and mortality occurred in the Middle East & North Africa (e.g., Qatar) and East Asia (e.g., South Korea), potentially driven by demographic aging, urbanization, and evolving diagnostic practices, though the relative contribution of each factor cannot be determined from these ecological data [24, 25, 26].

Attributable‐risk analysis shows that smoking, low calcium intake, and low dairy intake have modest associations with prostate cancer burden. Smoking is a well‐established risk factor for many cancers, including prostate cancer, via inflammatory and oxidative stress pathways [27]. However, its attributable fraction for prostate cancer is modest across all SDI levels, particularly in low‐SDI regions (< 3% of DALYs). Evidence regarding the inverse association between calcium or dairy intake and clinical outcomes is derived from comparative risk assessment models, which possess inherent limitations including residual confounding and measurement error [28, 29]. Therefore, while tobacco control and healthy diets are important for overall health, they should not be viewed as primary levers for reducing prostate cancer mortality, especially in low‐ and middle‐SDI regions. In these settings, the dominant drivers of high mortality are late‐stage diagnosis and limited access to curative treatments. Consequently, health‐system strengthening‐expanding diagnostic capacity, ensuring availability of surgery, radiotherapy, and androgen deprivation therapy, and improving cancer registration‐must take priority. In high‐SDI regions, where these health‐system factors are already well established, further tobacco control and dietary modifications may offer additional, albeit modest, benefits.

Age‐specific trends from 1990 to 2021 show that incidence rates increased in all age groups below 70 years, with the steepest rises in low‐middle SDI settings. This finding highlights age‑adapted screening strategies that consider the rising burden in younger populations, consistent with recent recommendations [30]. By 2021, the mortality peak had shifted to ages ≥ 85 years, reflecting prolonged survival. While elderly men in high‐SDI experienced marked mortality declines, rates continued to climb in low‐SDI nations, signaling successful chronic‐disease management in the former but persistent survival gaps in the latter. With global life expectancy projected to rise from 73.6 years in 2022 to 78.2 years in 2050 [31], population aging will further amplify prostate cancer burden, demanding adaptive policies to safeguard older adults' health.

The dual opposing pattern calls for region‑specific strategies. High‑SDI regions should prioritize reducing overdiagnosis and overtreatment by refining PSA screening and promoting active surveillance [17]. Low‑ and middle‑SDI regions, where mortality is highest and has shown little improvement, must focus on health‑system strengthening: expanding diagnostic access (PSA, biopsy, and pathology), building capacity for curative treatments (surgery, radiotherapy, and androgen deprivation therapy), strengthening cancer registration, and raising public awareness [7]. Tobacco control and dietary modifications should be secondary goals, as their attributable fractions for prostate cancer mortality are small. Tailoring strategies to each setting can reduce global burden while avoiding over‑intervention in high‑resource settings and under‑resourcing in low‑resource settings.

Several limitations merit mention. Reliable epidemiological data remain scarce in many middle‐ and low‐income settings, introducing considerable uncertainty. Variable uptake of PSA screening may also bias comparisons. COVID‐19 has added additional mortality uncertainty, and our risk‐factor analysis, reliant on existing literature, may omit important determinants.

Based on GBD 2021 data, prostate cancer incidence and mortality show a dual opposing pattern across SDI levels: incidence increases with SDI, mortality decreases. Low‑SDI regions have the highest mortality despite the fewest cases; high‑SDI regions face overdiagnosis. Attributable risk factors have modest associations, overshadowed by health‐system factors (screening, diagnosis, treatment access). Tiered strategies are therefore supported: (1) High‐SDI: refine screening, promote active surveillance; (2) Low‐/middle‐SDI: prioritize health‐system strengthening (cancer registration, diagnostic capacity, curative treatments, public awareness). Tobacco and dietary interventions are secondary. Tailoring policies to each context can achieve more equitable cancer control.

## Author Contributions

**Zhiping Ma:** conceptualization, data curation, funding acquisition, writing – original draft, writing – review and editing. **Cuicui Wang:** conceptualization, data curation, funding acquisition, writing – original draft, writing – review and editing. **Qin Zhang:** writing – original draft, data curation, formal analysis, project administration. **Zhongyu Lu:** writing – original draft, investigation, methodology, validation. **Dongsheng Chen:** investigation, writing – original draft, methodology, validation. **Xing Zhang:** conceptualization, data curation, funding acquisition, writing – original draft, writing – review and editing. **Yan Wang:** data curation, conceptualization, funding acquisition, writing – original draft, writing – review and editing.

## Funding

The authors have nothing to report.

## Disclosure

The corresponding author Yan Wang and Xing Zhang had full access to all of the data in this study and takes complete responsibility for the integrity of the data and the accuracy of the data analysis.

## Conflicts of Interest

The authors declare no conflicts of interest.

## Transparency Statement

The corresponding author Yan Wang, Xing Zhang affirm that this manuscript is an honest, accurate, and transparent account of study being reported.

## Supporting information


**Figure S1:** Geographic and socioeconomic classification of locations in the GBD study. (A) Clusters of 204 countries and territories. (B) 21 GBD regions. (C) Five SDI levels (low, low‐middle, middle, high‐middle, high). These classifications were used for all stratified analyses.

**Figure S2:** Temporal trends in age‐standardised rates of prostate cancer incidence and mortality by SDI level, 1990–2021. (A) Trends of ASIR. High‐SDI regions maintained the highest rates throughout but declined post‐2005, while low‐SDI, low‐middle SDI, and middle SDI showed steady increases, converging toward the global average. (B) Trends of ASMR. Global ASMR declined steadily, but low‐SDI regions showed little change, resulting in ASMR of low‐SDI surpassing high‐SDI by 2021.

**Figure S3:** Correlation between baseline burden and temporal change in prostate cancer rates. Scatter plots showing the relationship between 1990 age‐standardised rates and 1990–2021 EAPC. Left panel: ASIR versus incidence EAPC, indicating that regions with higher baseline incidence experienced slower subsequent increases or declines. Right panel: ASDR versus DALYs EAPC, showing similar patterns for disability‐adjusted life‐years. Bubble size represents 1990 case volume (> 50,000 cases in black).

## Data Availability

The data that support the findings of this study are available in the Global Health Data Exchange query tool l at https://vizhub.healthdata.org/gbd-results. These data were derived from the following resources available in the public domain: https://vizhub.healthdata.org/gbd-results.
