# Supplementary figures and images for: Global Dual Incidence‐Mortality Patterns of Prostate Cancer Across SDI Regions, 1990–2021: A Cross‐Sectional Study Based on Global Burden of Disease 2021
Source: Health Sci Rep. 2026 Jul 28;9(8):e72663. doi: 10.1002/hsr2.72663 (PMC13411467; doi:10.1002/hsr2.72663)

A

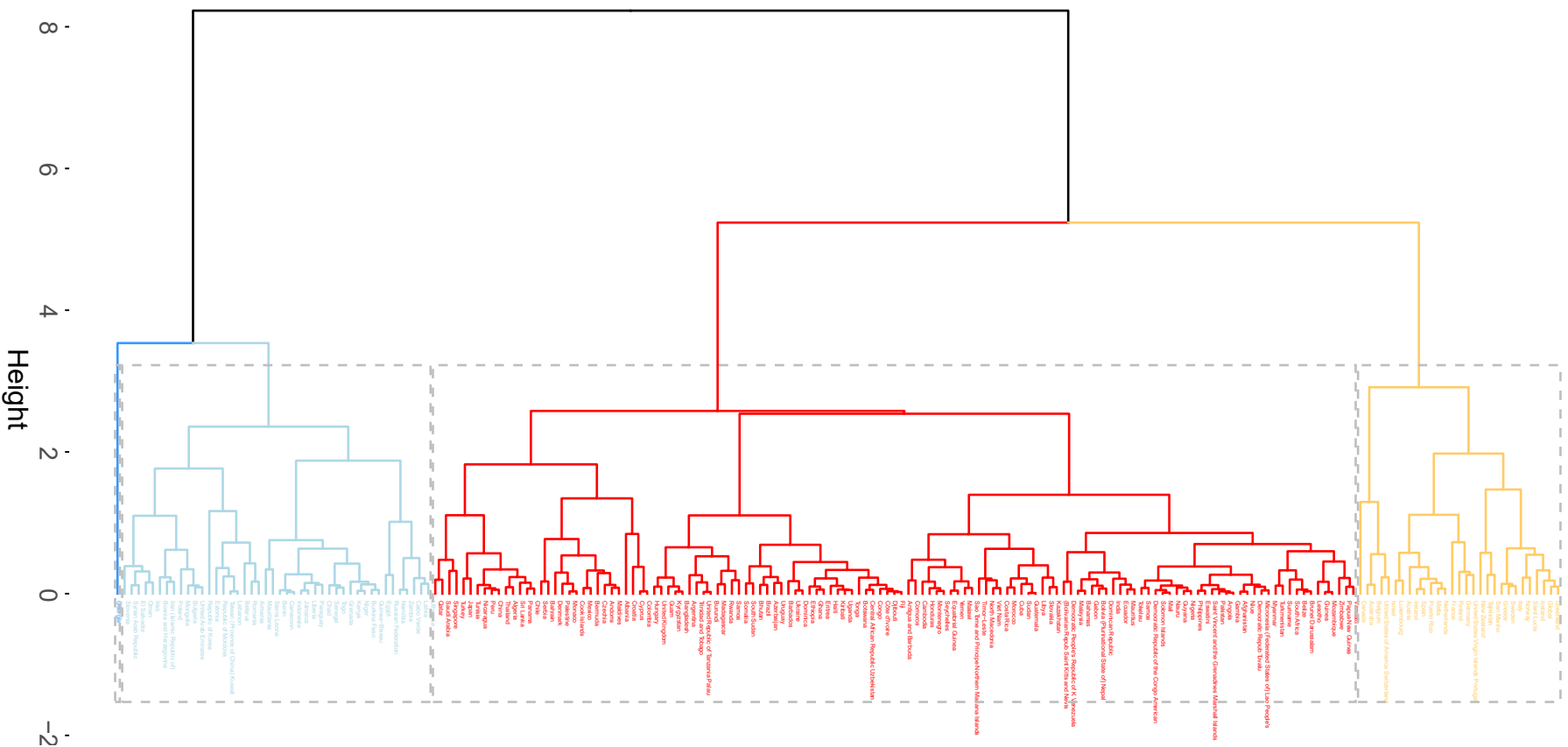

C

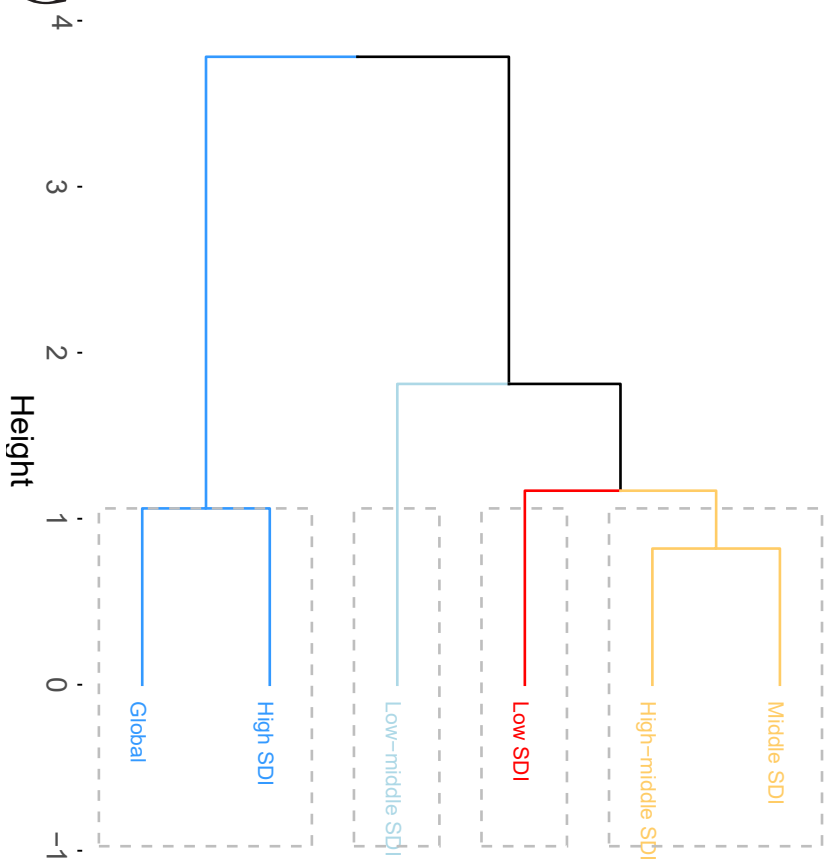

B

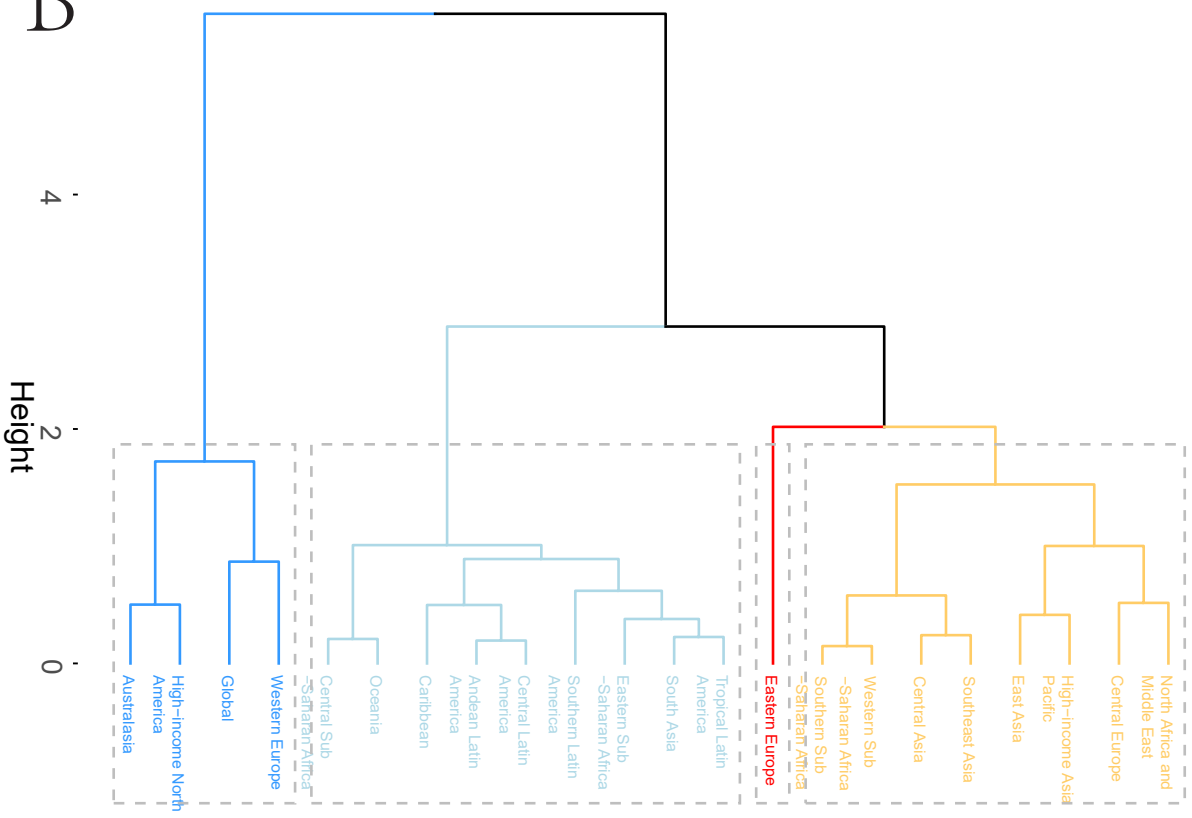

Supplement: Supplementary file 1 — Figure S1: Geographic and socioeconomic classification of locations in the GBD study. (A) Clusters of 204 countries and territories. (B) 21 GBD regions. (C) Five SDI levels (low, low‐middle, middle, high‐middle, high). These classifications were used for all stratified analyses. [file HSR2-9-e72663-s002.pdf]

A

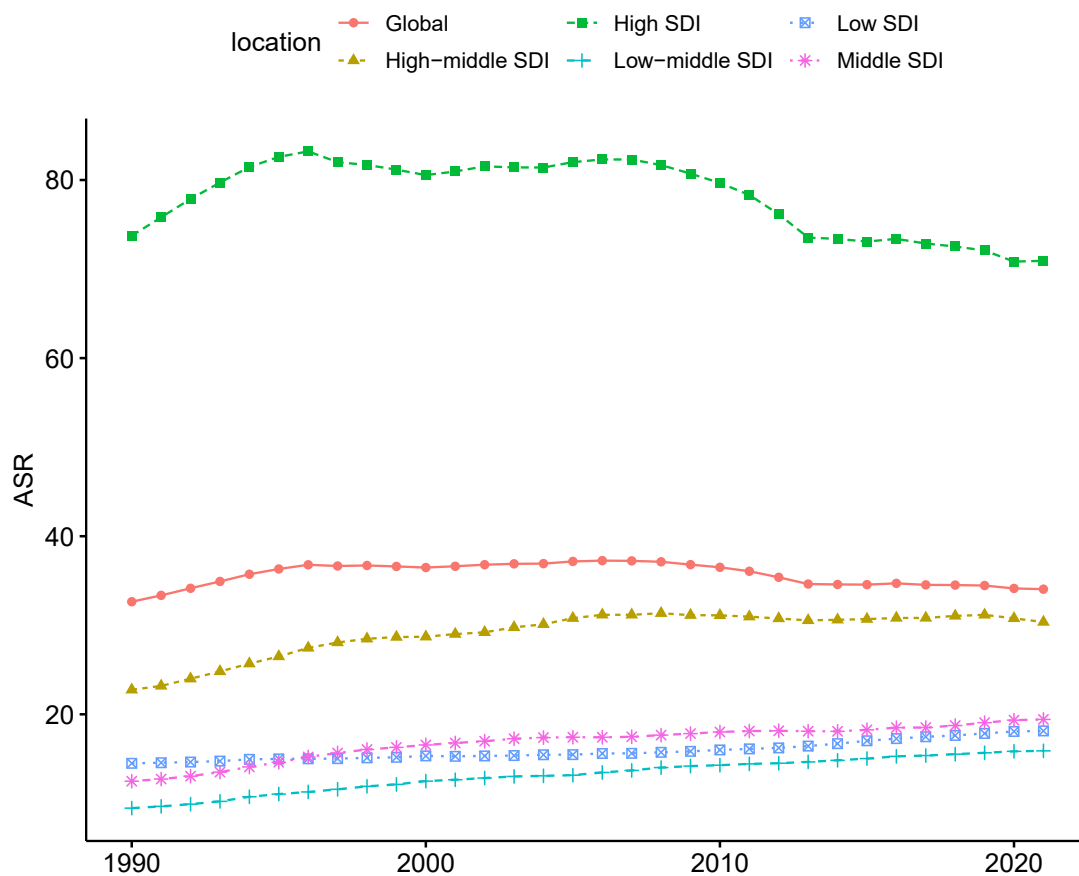

B

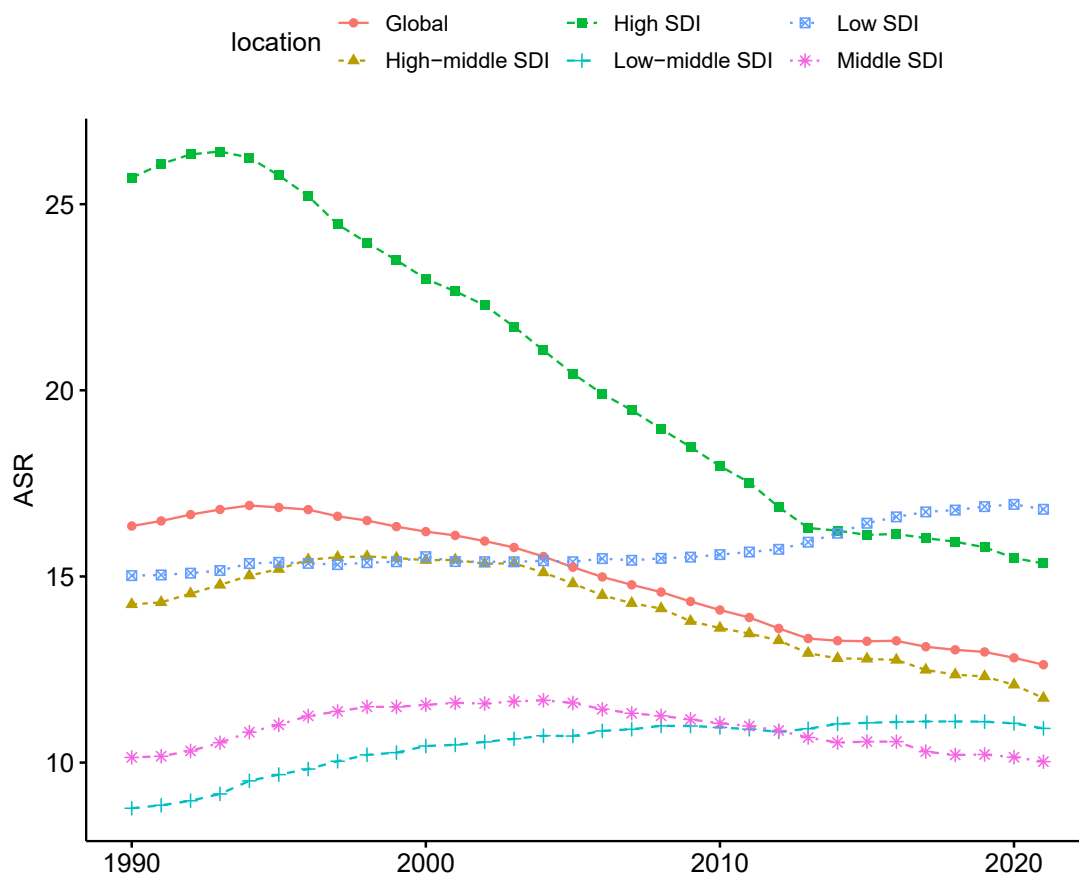

Supplement: Supplementary file 2 — Figure S2: Temporal trends in age‐standardised rates of prostate cancer incidence and mortality by SDI level, 1990–2021. (A) Trends of ASIR. High‐SDI regions maintained the highest rates throughout but declined post‐2005, while low‐SDI, low‐middle SDI, and middle SDI showed steady increases, converging toward the global average. (B) Trends of ASMR. Global ASMR declined steadily, but low‐SDI regions showed little change, resulting in ASMR of low‐SDI surpassing high‐SDI by 2021. [file HSR2-9-e72663-s001.pdf]

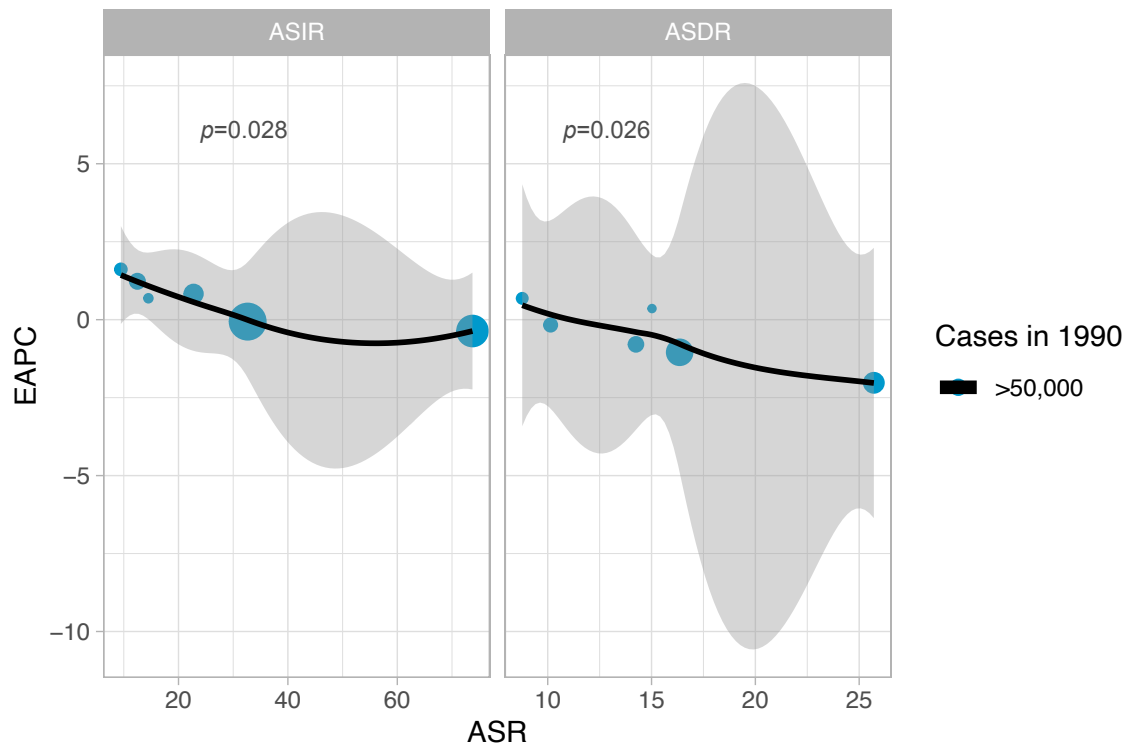

Supplement: Supplementary file 3 — Figure S3: Correlation between baseline burden and temporal change in prostate cancer rates. Scatter plots showing the relationship between 1990 age‐standardised rates and 1990–2021 EAPC. Left panel: ASIR versus incidence EAPC, indicating that regions with higher baseline incidence experienced slower subsequent increases or declines. Right panel: ASDR versus DALYs EAPC, showing similar patterns for disability‐adjusted life‐years. Bubble size represents 1990 case volume (> 50,000 cases in black). [file HSR2-9-e72663-s003.pdf]
